# A FAIR gold-standard benchmark for geographic entity recognition in cancer genomics literature for biomedical NLP

**DOI:** 10.1093/bioadv/vbag225

**Published:** 2026-08-27

**Authors:** Maricel G Kann, Karen O’Connor, Petra Tembei, Samuel Rosner, Graciela Gonzalez Hernandez

**Affiliations:** Biological Sciences Department, University of Maryland, Baltimore County, Baltimore, MD 21250, United States; Department of Biostatistics, Epidemiology, and Informatics, University of Pennsylvania, Philadelphia, PA 19104, United States; Biological Sciences Department, University of Maryland, Baltimore County, Baltimore, MD 21250, United States; Division of Oncology, University of Maryland Marlene & Stewart Greenebaum Comprehensive Cancer Center, Baltimore, MD 21201, United States; Department of Computational Biomedicine, Cedars Sinai Medical Center, Los Angeles, CA 90069, United States

## Abstract

**Motivation:**

Geographic biases in cancer genomics research limit precision oncology’s global applicability. Major consortia represent only 16 countries and sample less than 1% of 1.4 million publications worldwide. While automated literature mining could address these biases, the lack of benchmark datasets prevents rigorous development and validation of extraction models. We present a FAIR-compliant gold-standard benchmark of 129 manually curated cancer genomics studies spanning 46 countries, tripling geographic representation versus existing consortia. This dataset enables systematic evaluation of automated geographic information and molecular data extraction approaches and establishes a reference task for document-level geographic information extraction models.

**Results:**

Our benchmark highlights critical challenges for AI-powered literature mining. GeoBoost2 achieves 55% macro-F1 and 46% micro-F1 for patient-origin extraction across 129 articles, exposing precision–recall tradeoffs requiring domain adaptation. Seventy percent of studies include author affiliations from countries unrelated to patient cohorts, showing that metadata-based approaches systematically misattribute geographic provenance. Of 129 studies, 96 provide molecular data for 263 472 cancer patients, revealing substantial untapped literature-derived resources absent from major consortia and supporting training and comparison of domain-specific NLP models.

**Availability and implementation:**

The complete benchmark dataset, including manual annotations, automated extraction outputs, evaluation scripts, and supplementary documentation, is available at Zenodo with DOI 10.5281/zenodo.18259159.

## 1 Introduction

The biomedical literature represents a vast, underutilized data resource. PubMed indexes over 1.4 million cancer genomics publications spanning 188 countries, yet major consortia such as The Cancer Genome Atlas (TCGA) (The Cancer Genome Atlas Research Network *et al.* 2013), cBioPortal (Cerami *et al.* 2012), and the International Cancer Genome Consortium (ICGC) (The International Cancer Genome Consortium 2010) have systematically incorporated data from only 16 nations. This 99% underutilization reflects not just access barriers, but fundamental challenges in automated information extraction: published studies lack standardized, machine-readable geographic and molecular metadata, and existing natural language processing (NLP) tools lack domain-specific validation benchmarks. Without rigorously curated training datasets, developing robust extraction models remains intractable, a challenge generalizable across biomedical literature mining applications.

Major international consortia datasets predominantly originate from the United States and a small number of high-income countries, collectively representing less than 1% of the more than 1.4 million cancer genomics publications indexed in PubMed. This imbalance is compounded by substantial underrepresentation of racial and ethnic minorities. For example, Cheung *et al.* (2023) demonstrated that AACR Project GENIE (André *et al.* 2017) significantly underrepresents minority populations, and Spratt *et al.*’s (2016) analysis of TCGA has shown that 77% of samples are from White patients, with persistent gaps of Black, Hispanic, and Native American groups and global populations. Such demographic skew distorts biomarker discovery, limits generalizability of precision oncology findings, and obscures population-specific genomic variation.

Geographic underrepresentation also excludes regions where distinct environmental exposures, ancestry-related polymorphisms, and tumor biology shape disease risk and treatment outcomes. Carrio-Cordo *et al.* (2020) found that most published oncogenomic data originate from North America, Europe, and East Asia, with a near-complete absence of large-scale studies from Africa and other underserved regions. They also demonstrated that using author or submitter location as a proxy for sample origin often misattributes the geographic background of biosamples, underscoring the need for standardized, structured geographic metadata. Without incorporating molecular profiles that reflect the full geographic and ancestral spectrum of cancer biology, current precision oncology frameworks risk reinforcing existing disparities rather than mitigating them.

Recent advances in AI frameworks have expanded the potential for large-scale, automated mining of biomedical literature, but fully capturing global cancer diversity requires targeted innovation in AI and natural language processing (NLP) (Cheerkoot-Jalim and Khedo 2021, Cirillo *et al*. 2021, He *et al.* 2023). Transformer-based language models (Lee *et al.* 2020, Tinn *et al.* 2023), biomedical entity recognition (Sung *et al.* 2022), and relation extraction (Perera *et al*. 2020, Su *et al.* 2021)—aided by the advent of large language models (Chen *et al.* 2025) and generative AI- now enable precise identification of complex concepts such as gene-variant-disease relationships (Li *et al.* 2025), cohort attributes (Franklin *et al.* 2020), and outcomes, even from unstructured narratives or figures in the published literature (Hahn and Oleynik 2020, Zhao *et al.* 2021, He *et al.* 2023, Jin *et al*. 2024). However, these tools face critical domain adaptation challenges. Recent studies show that zero-shot and few-shot LLM prompting can be useful for biomedical NLP, but performance is inconsistent and often trails fine-tuned or task-specific models for structured extraction tasks such as NER and relation extraction. These limitations reinforce the need for curated annotated resources that support supervised training and domain-specific evaluation rather than relying on generic genAI prompting alone (Chen *et al.* 2025, Miao *et al.* 2025). Geolocation extractors that work well on viral sequence metadata or news articles may lose precision in scientific literature, where geographic mentions may reference author institutions, conference locations, or unrelated citations rather than patient cohort origins (Magge *et al.* 2020). The classic precision-recall tradeoff in automated information extraction becomes particularly acute in biomedical contexts, where false positives undermine data quality and downstream analyses. Addressing these challenges requires benchmark datasets with expert-level manual curation to train and evaluate domain-specific models, resources currently absent from the literature mining ecosystem. From a bioinformatics perspective, accurate geolocation of patient cohorts is a critical upstream step for integrating literature-derived molecular profiles with genomic databases, exposure resources, and clinical registries. However, no standardized benchmark currently exists to support method development or to compare NER and geolocation pipelines in this setting. Our work fills this gap by defining a concrete extraction task, providing a curated evaluation dataset, and establishing baseline performance for a widely used geolocation pipeline, thereby enabling systematic method comparison and future incorporation of large language models.

Here, we establish methodological foundations for geolocation-aware literature mining through development of a FAIR-compliant benchmark dataset. Our manually curated gold standard comprises 129 cancer genomics studies representing 46 countries, tripling geographic coverage relative to major consortia. The benchmark was constructed through independent, dual-curated document-level extractions across four data domains, with discrepancies resolved by a third curator to establish the gold standard. We systematically evaluate GeoBoost2 (Magge *et al.* 2020), a state-of-the-art geolocation tool, exposing fundamental precision-recall tradeoffs in automated extraction. Beyond benchmarking, we demonstrate the dataset’s utility: 96 studies provided molecular data for 263 472 patients, revealing population-specific variants absent from existing databases. Our reusable curation protocol, annotation schema, and benchmark dataset enable training of next-generation extraction models while providing an immediate application in addressing cancer genomics equity. This work advances both data science methodology and domain science by providing concrete infrastructure for systematic literature mining at scale.

## 2 Methods

### 2.1 The data collection

We conducted two separate comprehensive searches of PubMed to identify studies containing genomic information related to cancer. The first search encompassed all cancer types and the second was focused on lung cancer. Our searches consisted of two facets: (i) disease specific terms (either ‘cancer’ or ‘lung cancer’) and (ii) genomic related terminology in the title, abstract or MeSH terms. Our search dates were limited to January 1st 2000, when earliest genomic studies started, to November 1st 2024 (present). The complete search strategy is detailed in Supplementary File 1.

From the lung cancer literature collection, we created a third dataset (‘LungCancer-OA’), consisting of articles in the PubMed Central Open Access subset (PMC-OA). The PMC-OA is a collection of articles from PubMed Central available in a standardized machine-readable format which facilitates their use for text mining and AI research. For all articles in the lung cancer dataset, we utilized the NCBI PMC-OA API to identify articles available in the PMC-OA. This search resulted in 3216 articles.

### 2.2 Data selection

We selected 300 articles from the LungCancer-OA collection, representing about 10% of the dataset, for manual curation and automated extraction of study participant location. Our sampling strategy was designed to support a representative benchmark and to characterize geographic bias in the accessible literature without artificially inflating country representation. To analyse the relationship between study participant locations and researcher affiliations, we first extracted author affiliation locations from all articles. For each article in the LungCancer-OA collection, the article’s metadata was extracted from PubMed using the Pubdata2XL tool. We included only studies with authors from two or more different affiliation countries. The final sample (*n* = 300) was selected using weighted random sampling based on the frequency distribution of affiliation countries to ensure all countries were represented in the final dataset.

Curators then manually reviewed all 300 articles, excluding those not cancer-related, lacking genomic data, or missing patient location information. This process yielded a final benchmark dataset of 129 manuscripts. While our curated set represents a focused sample, it provides sufficient statistical power for our primary analyses and establishes a robust benchmark for training automated extraction models. The sample size aligns with established practices in literature mining validation studies and, though only ∼0.1% of the total cancer genomics literature, enables detailed manual verification while maintaining representativeness.

### 2.3 Data curation

Data extraction was performed using Covidence (n.d), an online platform for data curation. Curators were presented with a standardized extraction form with the annotation types to be collected from each article. In addition to the location of participants included in the study, curators extracted other relevant information from each study, including disease information, cohort size and patient demographics, molecular data, whether research was related to a clinical trial, and reported information to linked repositories for the data (Table 1). The complete data extraction form is presented in Supplementary File 2.

**Table 1:** Data extraction fields with brief description.

Section	Field	Description
Inclusion Check^a^	Disease Studied	Disease focus of the study—Lung Cancer, Other Cancer (with name), Other Disease (not cancer related)
	Human Subjects Research	Study included human participants or used data from human participants (Yes/No).
Characteristics of Included Study	Clinical Trial	Study is a clinical trial (Yes/No); if Yes, record the clinical trial registration number
	Aim/focus of study	Primary aim(s) or focus of the study—diagnosis, treatment, outcomes, genomics, or other
	Number of Participants	Total size of the study cohort
	Number of Cohorts	Number of distinct cohorts in the study
Geographic Location of Participants (Country level)^a^	Geographic information of participants	Geographical information about the participants reported (Yes/No)
	If Yes, section in paper where geographical information reported	Categorical selection of location(s) within the paper where geographic information appears^b^ (supporting sentence(s) extracted)
	Multiple participant locations	For studies with multiple participant locations: country name and number of participants per country
Demographic Information of Participants	Demographic information	Presence or absence of demographic data—age, sex/gender, race/ethnicity/nationality. ‘No’ selected if none reported.
	Demographic information location (section within paper)	Categorical selection of location(s) within the paper where demographic information appears^b^
	Classification of demographic reporting level	Individual patient or as aggregate/summary statistics
Genomic Sequencing and Mutation Data	Mutation data	Mutation data for participants is reported (Yes/No). If No, no further mutation fields are completed
	Mutation data location (section within paper)	Categorical selection of location(s) within the paper where mutation data appears^b^
	Classification of mutation reporting level	Individual patient level or as aggregate/population-level data
	Genomic sequencing data (Does the study include any genomic sequencing data?)	Whether any genomic sequencing data for participants is reported (Yes/No). If No, no further genomic fields are completed
	Genomic data location (section within paper)	Categorical selection of location(s) within the paper where genomic sequencing data appears^b^
	Classification of genomic reporting level	Reported at the individual patient level or as aggregate/population-level data
	Database/Repository Information	Data deposited in an external repository (Yes/No); if Yes, record repository name and any associated identifier (e.g. accession number)

All data extracted are available in the gold standard benchmark.

a If Disease Studied, Human Subjects, or Geographic Location were negative, no extractions were completed.

b Options—text, tables, figures, and/or Supplemental Material.

Curators were trained using guidelines developed for the task (Supplementary File 3). For each article, two curators independently extracted the data. A third curator performed the consensus, resolving any disagreements and finalizing the extracted data to establish the gold standard.

Geographic locations were extracted at the country level. After consensus, all locations were standardized to the GeoNames database (GeoNames n.d) and cancer types were normalized to the National Cancer Institute thesaurus (NCIt) (National Cancer Institute n.d). Annotations are provided at the document level, capturing structured metadata fields rather than inline text spans. The benchmark is therefore suited for evaluating document-level information extraction systems rather than mention-level NER pipelines.

Inter-rater reliability was assessed *post hoc* for 9 closed-ended categorical fields using Cohen’s *κ* (Table 2). Annotation followed a sequential screening cascade: all 294 double-annotated papers were assessed for cancer type studied (*κ* = 0.765), papers passing this screen were assessed for human subjects inclusion in study (*n* = 250, *κ* = 0.59). Next the papers were assessed for whether geographic locations were reported in the study (*n* = 157, *κ* = 0.47). Due to the screening instructions provided to curators to discontinue extraction if the paper did not pass on of the screening questions, only papers that passed the screen by both annotators were included in the subsequent round for IAA calculation. The remaining papers were assessed for all extraction fields (*n* = 82). Mean *κ* across all 9 fields was 0.482 (range 0.30–0.77), reflecting fair to substantial agreement (Viera and Garrett 2005). Per field reporting provided in Table 2 provides a more informative characteristic of annotation difficulties than an aggregate score alone (Elangovan *et al.* 2024). Screening fields showed higher agreement (*κ* = 0.473–0.765), consistent with their more objective criteria. Extraction fields requiring interpretation of heterogeneous reporting practices showed lower agreement (*κ* = 0.299–0.621), which is expected given the variability in how geographic, demographic, and molecular data are reported across biomedical literature. All disagreements were resolved by a third curator who established the final gold standard.

**Table 2:** Inter-annotator agreement for categorical extraction fields.

Data field	Annotation phase	*n*	Cohen’s *κ*	Interpretation
SCREENING FIELDS				
Cancer type studied	Screening 1	294	0.765	Substantial
Human subjects reported	Screening 2	250	0.586	Moderate
Geographic location reported	Screening 3	157	0.473	Moderate
EXTRACTION FIELDS				
Clinical trial	Extraction	78	0.621	Substantial
Aim/focus of study	Extraction	76	0.299	Fair
Demographic information reported	Extraction	76	0.361	Fair
Genomic mutation information	Extraction	81	0.451	Moderate
Genomic sequencing data	Extraction	80	0.299	Fair
Genomic data deposited in repository	Extraction	41	0.481	Moderate

### 2.4 Automated location extraction

We performed automated location extraction from the 300 papers using a previously validated system, GeoBoost2. The details of how the system works was described in Magge *et al.* (2020). Briefly GeoBoost2 is a natural language processing pipeline designed to extract location mentions from biomedical literature using deep learning and machine learning methods. For each of the 300 papers, the title, abstract, text, tables, table captions and figure captions, from the BioC XML (Comeau *et al.* 2013) format downloaded from PMC-OA, was provided as input for GeoBoost2 excluding author affiliation fields from the input. The extracted location is resolved and normalized to standardized location names using the GeoNames database. While originally designed for viral sequence geolocation, we evaluated its generalizability to other domains by comparing its performance against the manually curated dataset.

### 2.5 FAIR principles implementation

The benchmark dataset follows the FAIR (Findable, Accessible, Interoperable, Reusable) principles. Findability is ensured through depositing in Zenodo with a registered DOI-registered, and rich identifiers including PubMed identifiers. Accessibility is supported by open repository sharing under a permissive CC-BY 4.0 license with detailed provenance to support robust reuse and extension. Interoperability is achieved through distribution in multiple, standard machine-readable formats (e.g. tabular and XML). All papers are linked to their unique PubMed identifier. The extracted locations are standardized to the GeoNames database (GeoNames n.d). All cancer types extracted are normalized to the NCIt (National Cancer Institute n.d). All extracted clinical trial information and consortia database information include the accession or identification numbers linked to their respective database. Reusability is facilitated by the comprehensive curation documentation (Supplementary Files 2 and 3), annotation guidelines, and evaluation scripts enabling reproduction of analysis and the extension of the benchmark beyond the baseline.

## 3 Results

### 3.1 Geographical analysis of cancer genomic literature

**Figure 1:**
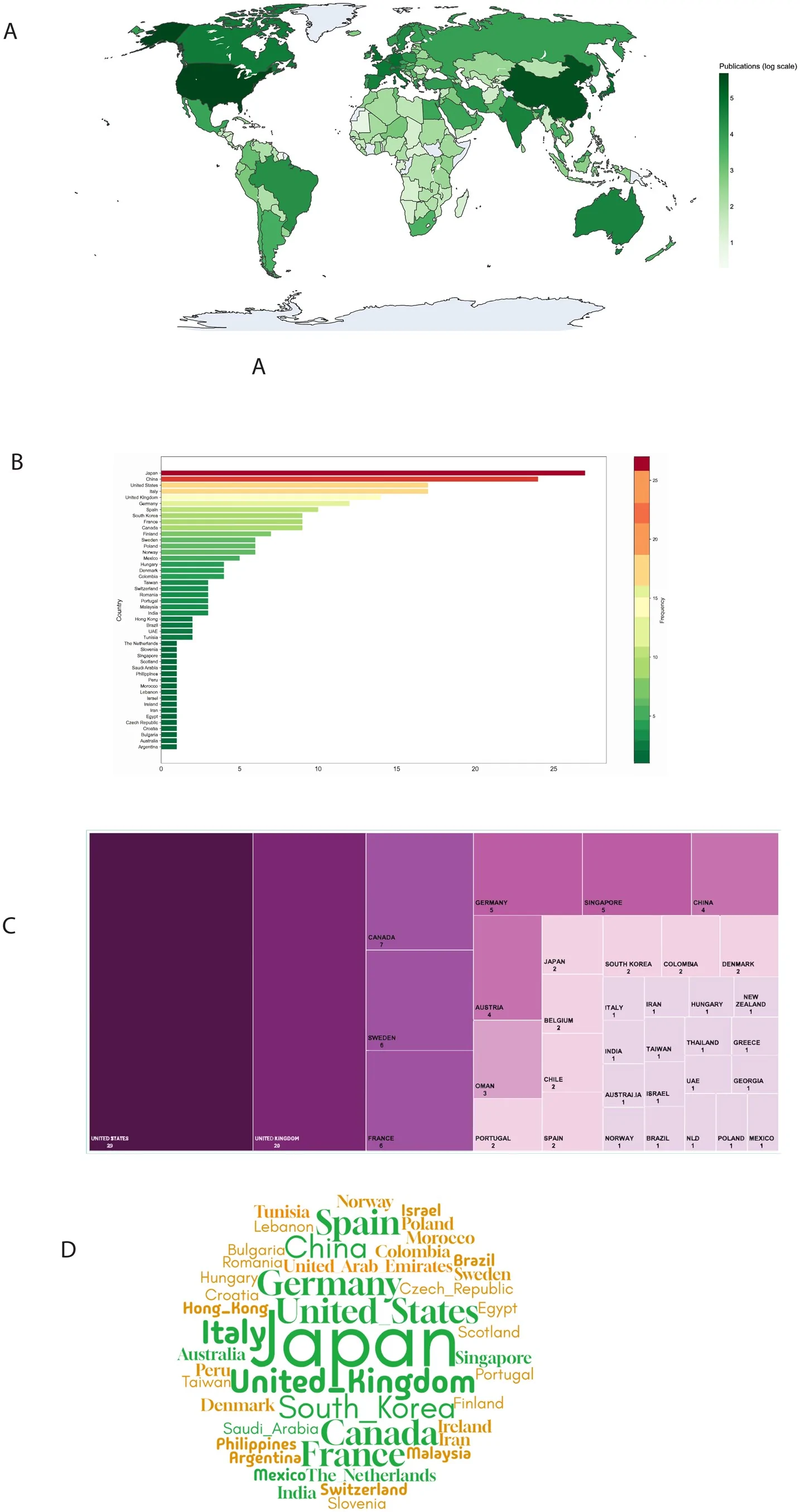
Global geographic representation in cancer genomics research. (A) World map showing author affiliation locations from 1.4 million cancer genomics publications across 188 countries, used as a proxy for global research distribution. (B) Geographic distribution of patient populations from 46 countries represented in the manually curated gold standard dataset of 129 studies. (C) Visualization of mismatches between author affiliation countries and actual patientocohort locations, highlighting studies where affiliations do not reflect true research sites. (D) Gold standard geographic distribution (country size proportional to article frequency). Green indicates countries in both the benchmark and major consortia, revealing bias toward high-income nations.

To validate our literature mining approach and characterize the geographic representation of published cancer genomics research, we analysed PubMed using two tailored search queries: a general cancer genomics query retrieving 1.4 million manuscripts and a lung cancer-specific query yielding 143 000 manuscripts (Supplementary File 1). Figure 1A illustrates the distribution of author affiliations from 1.4 million cancer genomics publications mapped to 188 countries, using affiliation locations as a proxy for research activity rather than actual patient cohort origins. To establish a benchmark dataset, we screened an initial set of 300 randomly selected publications from the PubMed Central Open Access from the lung cancer specific query. We used lung cancer as the initial corpus because it provided a large, accessible open-access set of articles suitable for manual curation and benchmark development; however, the resulting annotation schema is cancer-agnostic and can be extended to other malignancies in future work. Applying inclusion criteria (cancer-related, genomic data present, patient location data available), we retained 129 manually curated publications as our gold standard benchmark (see Fig. S1 for screening flowchart). Among these 129 studies, 107 (83%) focused on lung cancer, while 22 (17%) addressed other malignancies.

### 3.2 Geographical representation and affiliation discrepancies

The benchmark dataset revealed patient populations from 46 distinct countries across the 129 studies (Fig. 1B). This geographic diversity within our manually curated set demonstrates the value of systematic literature mining for expanding data representation. Importantly, 11 studies (9%) reported research conducted in countries unrepresented in author affiliations, revealing limitations of affiliation-based metadata extraction (Table S1). Conversely, 90 studies (70%) included author affiliations from 35 countries unrelated to the actual research locations, a systematic bias with implications for automated geolocation models. The United States accounted for 32% of these mismatches (*n* = 29/90 studies), followed by the United Kingdom (22%, *n* = 20/90 studies). These affiliation-origin discrepancies highlight why domain-specific geographic entity recognition models are essential: generic extraction tools cannot reliably distinguish patient cohorts from institutional affiliations without contextual disambiguation (Fig. 1C).

### 3.3 Automated versus manual geographical tagging

We systematically evaluated GeoBoost2 (GB2) (Magge *et al.* 2020), a state-of-the-art geographic entity recognition tool, against our manually curated gold standard using both micro and macro averaging metrics across 129 articles (Table S2).

*Per-article (macro) performance* showed moderate precision (35.3%) but high recall (76.2%), yielding an F1 score of 55.3%. This indicates GB2 identifies most articles containing patient origin countries but struggles with precision at the article level, averaging one false positive per correctly identified article.

*Per-country (micro) performance* across all 449 country mentions revealed even greater precision challenges: 32.9% precision (TP = 133, FP = 271, FN = 45), 74.7% recall, and 45.7% F1. Large articles with multiple countries disproportionately influence micro scores, while macro averaging reveals consistent per-article performance regardless of article size.

These results confirm GB2 achieves high sensitivity (79% of articles have ≥1 correct country) but suffers 89% false positive rate at the article level, with only 7% of articles (9/129) receiving perfectly clean extractions. The precision-recall gap reflects fundamental domain adaptation challenges: GB2 cannot disambiguate patient cohort origins from the 70% prevalence of unrelated institutional affiliations in our benchmark, underscoring the need for biomedical-specific training data.

### 3.4 Consortium-driven geographic biases

To validate the utility of literature mining for expanding geographic coverage, we analysed eight major cancer genomic databases [TCGA (The Cancer Genome Atlas Research Network *et al.* 2013), cBioPortal (Cerami *et al.* 2012), AACR GENIE (André *et al.* 2017), COSMIC (Tate *et al.* 2019), ICGC (The International Cancer Genome Consortium 2010), GDC (Grossman *et al.* 2016), UCSC Xena (Goldman *et al.* 2020), OncoDB (Tang *et al*. 2022)]. Despite aggregating data from 455 studies (cBioPortal), 25 000+ manuscripts (COSMIC), and 65 projects (GDC), these eight resources collectively represent only 16 countries (Supplementary Table 3), compared to the 46 countries in our benchmark dataset. This disparity persists despite global platform reach: 70% of TCGA/GDC data originates from U.S.-led consortia, and ICGC’s landmark publications primarily reflect high-income nation contributions (Fig. 1D). These findings demonstrate that literature mining systematically captures geographic diversity unavailable through consortium-centric approaches, validating the complementary value of our benchmarking approach.

### 3.5 Data extraction and study metrics

**Figure 2:**
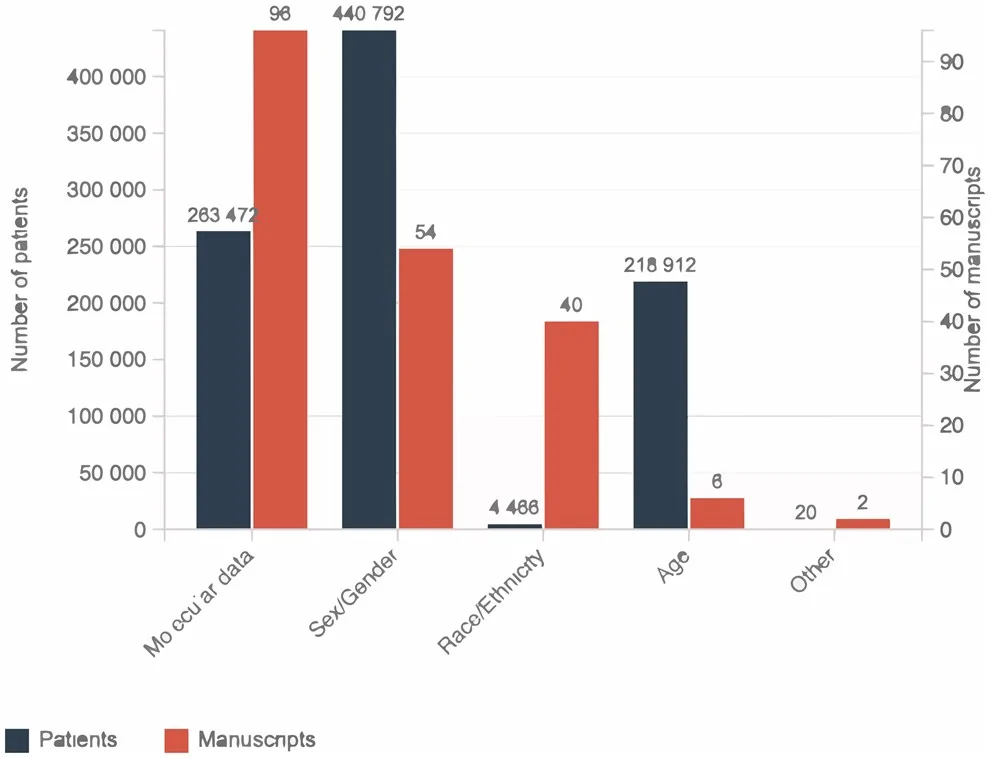
Distribution of patient-level data types in the gold standard set. Bar plots showing the number of patients and manuscripts reporting molecular data, race/ethnicity, sex/gender, age, and other demographic features among the 129 curated studies. Molecular data were available for 263 472 patients (across 96 manuscripts); demographic variables varied in completeness across the cohort.

**Figure 3:**
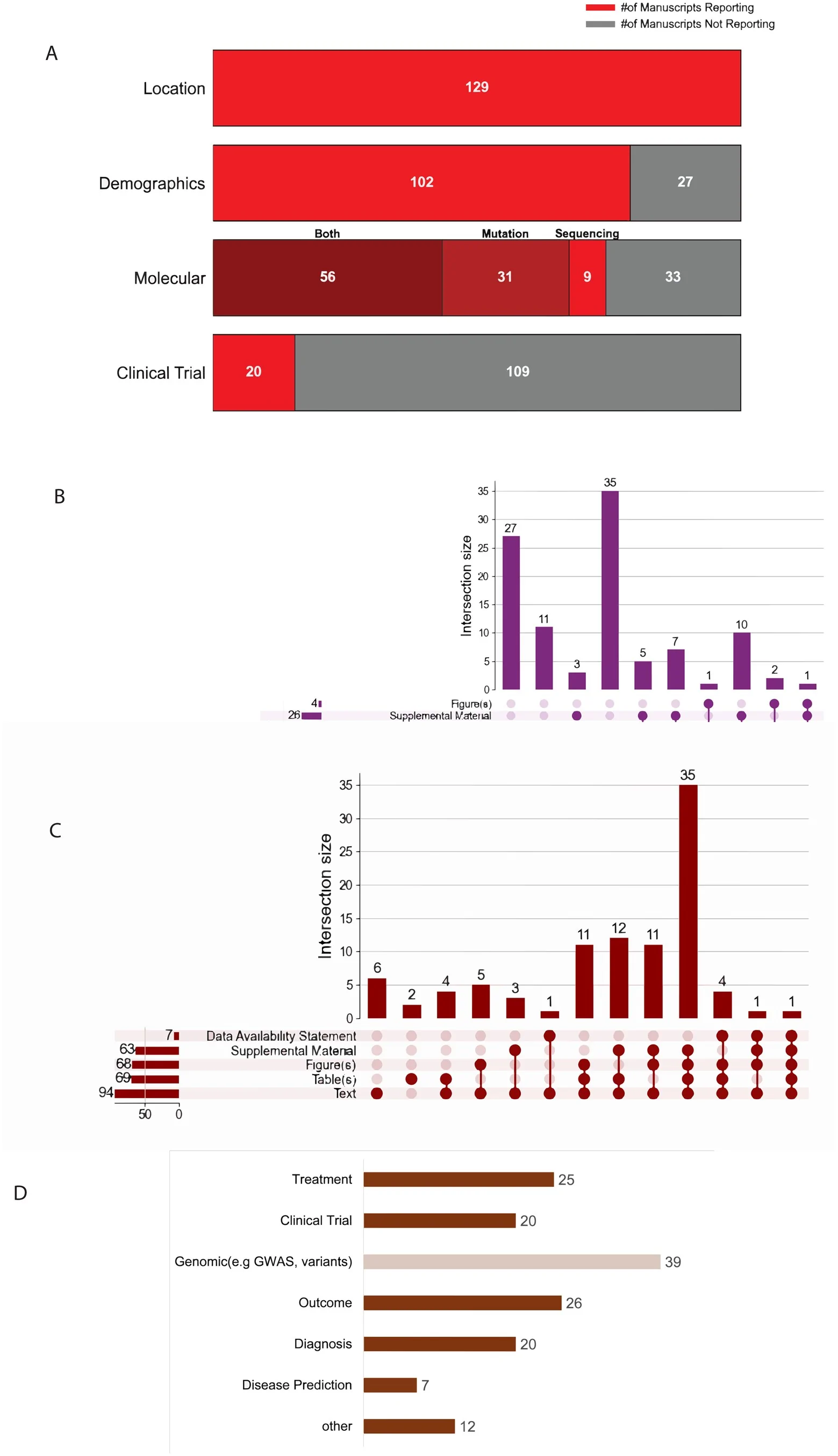
Patterns of data reporting and study aims in the curated dataset. (A) Reported data types (location, demographics, molecular data, clinical trial participation) across 129 manuscripts. (B) Locations within manuscripts where demographic data are reported (main text, tables, figures, supplementary materials). (C) Locations within manuscripts where molecular data are reported. (D) Primary research objectives and study aims identified in the curated literature, including treatment, clinical trial participation, genomics, outcomes, diagnostics, disease prediction, and other categories.

Our systematic extraction assessed four primary data types across the 129-study benchmark: patient geographic location, demographic characteristics, clinical trial documentation, and molecular profiling (genomic sequencing and mutation data) (Table 1). The composition and completeness of these data types are presented in Fig. 2. Geographic information was available in all studies (100%), appearing primarily in cohort description and Methods sections. Demographic details, including age, gender, or race/ethnicity, were reported in 79% (*n* = 102/129) of manuscripts, covering 719 531 patients. Only 16% (*n* = 20/129) of studies explicitly documented clinical trial participation. Molecular characterization data, encompassing mutational and genomic sequencing results, appeared in 74% (*n* = 96/129; Fig. 3A) of manuscripts. Notably, the 96 studies providing molecular data included a total of 263 472 cancer patients for whom this information was available, representing a large and diverse cohort that exceeds the combined coverage of major cancer genomics consortia. This composition demonstrates the untapped scale of molecular data accessible through systematic literature mining.

**Figure 4:**
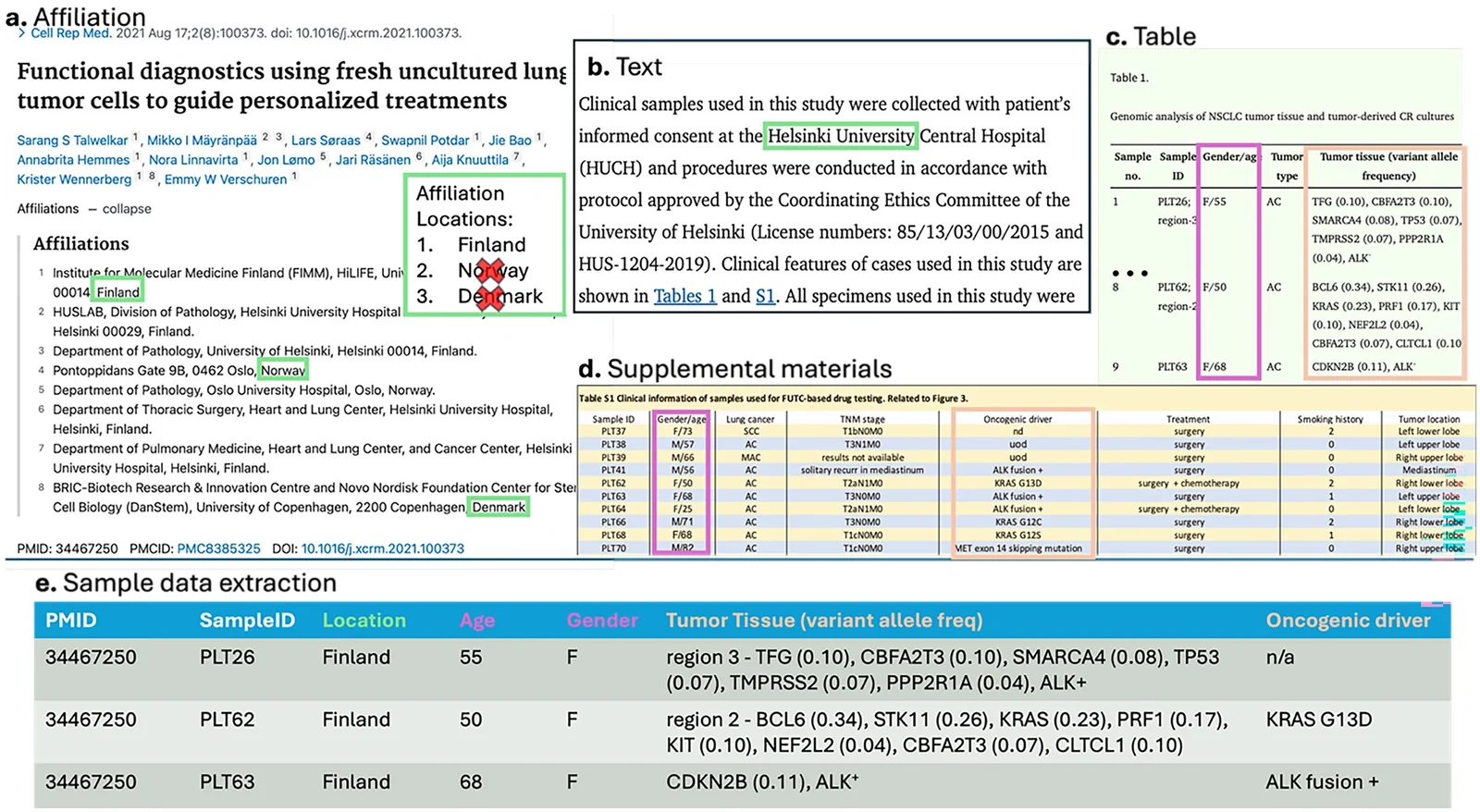
Example of data sources within a cancer genomics article. This figure is a representative illustration of the locations and other types of data included in a single publication: (a) author affiliations in article metadata, (b) specific cohort location in the main text, (c) individual patient data in tables, and (d) supplemental information. The main text provides the most accurate location—samples were all collected in Finland—whereas relying on metadata would have led to erroneous attribution. (e) Highlights how fully automated extraction can integrate patient-level data with consortia datasets to enhance cancer genomic resources.

Molecular data were further stratified by granularity and type. Genomic sequencing results were included in 50% of studies (*n* = 65/129), with the majority (88%, *n* = 57/65) reporting aggregate population-level data and a minority (12%, *n* = 8/65) providing individualized patient-level results. Mutation data were reported in 67% of studies (*n* = 87/129), predominantly as grouped population findings (87%, *n* = 76/87), and less frequently as patient-specific variants (13%, *n* = 12/87).

### 3.6 Data reporting heterogeneity: implications for extraction pipeline design

Demographic information for 719 531 patients was reported in 79% of studies (*n* = 102/129). Of these, 97% (*n* = 99/102) presenting these data in the main text and tables as aggregated summaries (Fig. 3B), while only 3% reported exclusively in Supplementary Materials. Sex and gender information appeared in 42% of studies (*n* = 54/129), with 76% (41/54) in main text only and 22% (12/54) in combined locations. This heterogeneous reporting distribution reflects the variability in manuscript preparation standards across biomedical literature.

Molecular data reporting showed similar diversity: 74% of studies (*n* = 96/129) included molecular findings with 98% (94/96) presenting information in main text and tables, 65% (*n* = 63/96) supplemented these with additional data in Supplementary Files, and 70% (68/96) embedding results in figures (Fig. 3C). This multi-modal distribution of information across text, tables, figures, Supplementary Files, creates challenges for text-only extraction pipelines and necessitates integrated approaches combining NER, table parsing, and image analysis for comprehensive data capture. Figure 4 illustrates this heterogeneity within a representative cancer genomics article: author affiliations, cohort-location statements in the main text, patient-level tables, and supplementary files provide complementary but non-equivalent evidence, with the main text correctly identifying Finland as the sample-collection location. Notably, only 12% (*n* = 15/129) deposited raw genomic data in external repositories such as the European Genome-Phenome Archive (EGA) (Lappalainen *et al.* 2015) and Gene Expression Omnibus (GEO) (Edgar *et al.* 2002), and crucially, zero of the 96 studies with molecular data contributed to major cancer consortia, indicating substantial untapped resources for consortium expansion and demonstrating why literature-driven approaches complement consortium curation.

### 3.7 Study aims and research landscape of the benchmark

The 129 studies in our benchmark dataset pursued diverse objectives, as summarized in Fig. 3D, which enables evaluation of extraction approaches across heterogeneous research contexts. Foundational research predominated, with 63% (*n* = 81/129) aiming to characterize molecular landscapes of specific cancers within defined populations or to identify novel biomarkers. Translational and clinical aims included linking genomic alterations to clinical outcomes (28%, *n* = 36/129), evaluating targeted therapies (19%, *n* = 25/129), and investigating genetic drivers of exceptional treatment responses (9%, *n* = 12/129). This thematic diversity ensures the benchmark captures geolocation and molecular data extraction challenges across multiple research domains, from discovery to translational science, enhancing the utility of this gold standard for training robust, generalizable extraction models applicable to the full spectrum of cancer genomics literature.

## 4 Discussion

### 4.1 A FAIR-complaint benchmark for training geolocation extraction models

The absence of domain-specific training data represents a fundamental bottleneck in biomedical literature mining. While transformer-based models like BioBERT and large language models demonstrate impressive capabilities on generic named entity recognition (NER) tasks (Lee *et al.* 2020, Tinn *et al.* 2023), their performance degrades substantially when applied to specialized biomedical contexts requiring fine-grained semantic distinctions. Our manually curated benchmark of 129 cancer genomics studies addresses this gap by providing expert-annotated geographic entity labels with contextual role assignments (patient origin versus institutional affiliation versus incidental mention). This resource enables supervised training of domain-specific models, directly addressing the precision-recall tradeoff we quantified through systematic GeoBoost2 evaluation. Unlike existing biomedical NER benchmarks focused on gene mentions [BioCreative (Smith *et al.* 2008)], disease entities [NCBI Disease Corpus (Doǧan *et al*. 2014)], or clinical concepts [i2b2 challenges (Uzuner *et al.* 2011)], our dataset targets geographic information extraction, a crosscutting annotation task relevant across disease domains, environmental health, and global health equity research. The benchmark’s FAIR compliance ensures machine-readability (interoperable formats), persistent accessibility (repository deposition with DOI), and explicit reuse permissions (open license), facilitating training data integration into NLP pipelines across the research community. Compared with the GeoBoost (Tahsin *et al.* 2018) and GeoBoost2 (Magge *et al.* 2020), which are natural language processing pipelines developed to extract and normalize locations of infected hosts from virus-related GenBank records and linked articles for phylogeographic metadata enrichment, our work introduces a manually curated benchmark corpus derived from cancer genomics literature, with document-level annotations of patient geographic origin and molecular profiling information. This difference in source material (virus sequence repositories versus cancer genomics articles), annotation granularity, and intended use makes our resource complementary to prior geolocation work and better suited for evaluating biomedical literature–mining methods in oncology.

Our dataset’s focus on lung cancer (83% of studies) reflects both our targeted search strategy and lung cancer’s prominence in precision oncology research. While this may limit direct generalizability to other cancer types, lung cancer’s molecular diversity and global disease burden make it an appropriate model system for demonstrating geographic biases. Future work should expand to other cancer types to validate broader applicability. Although the current benchmark is enriched for lung cancer, its annotation schema, curation protocol, and file formats are cancer-agnostic, and the same framework can be directly extended to additional cancer types and other disease domains.

### 4.2 Quantifying domain adaptation challenges through systematic evaluation

Our benchmark evaluation of GeoBoost2 as a baseline system quantifies the fundamental challenge of cross-domain transfer in geographic entity recognition. The tool’s 79% sensitivity coupled with 89% false positive rate exemplifies the classic precision-recall tradeoff: models optimized for high recall in generic contexts (news articles, viral sequence metadata) sacrifice precision when confronted with biomedical literature’s specialized discourse patterns. The core challenge lies in contextual disambiguation. Scientific articles contain multiple geographic entity types serving distinct semantic roles: patient cohort origins (target entities), author institutional affiliations (metadata), conference locations (citations), and comparative population references (discussion). Generic NER models lack the contextual understanding to distinguish these roles, treating all location mentions identically. The systematic false-positive flagging of the United States in 69% of studies, despite no patient cohort linkage, reveals how institutional affiliation frequencies in author metadata contaminate automated extraction.

Addressing this requires models that learn contextual patterns through domain-specific training. Promising approaches include: (i) multi-task learning architectures jointly predicting entity types and semantic roles, (ii) attention mechanisms leveraging document section structure (Methods sections more likely contain patient origin descriptions), and (iii) relation extraction models linking geographic entities to patient cohort mentions. The benchmark is also well suited for evaluating emerging large language models and instruction-tuned systems on biomedical geolocation tasks, enabling direct comparison with automated extraction pipelines under consistent conditions. Our benchmark’s dual annotation of both geographic entities and author affiliations enables training such sophisticated architectures, providing positive examples (patient origins) and contrastive negative examples (institutional affiliations) essential for disambiguation learning. These findings generalize beyond geographic entities: similar disambiguation challenges affect drug name extraction (mentioned versus prescribed), gene mention detection (studied versus background), and adverse event identification (reported versus hypothesized). Domain adaptation through specialized training data represents a generalizable solution pathway across biomedical NLP tasks.

### 4.3 Limitations and future directions

This benchmark has several limitations that should be considered when interpreting results and planning future extensions. First, annotations were performed at the document level, associating geographic entities, participant demographics and molecular data with the article as a whole rather than linking them to specific sentences, passages or figures. While sufficient for evaluating extraction at the study-participant level, this document-level approach does not capture the intra-document structural context needed to train fine-grained, span-level extraction models. Future iterations of this benchmark should therefore incorporate span-level annotations to support more precise NER and relation extraction training.

Second, not all extracted entities were normalized to controlled vocabularies or ontologies. Geographic entities were resolved to GeoNames identifiers, cancer types to NCIt, and extracted clinical trial and consortia information includes accession or other database identifiers; however, demographic categories were not systematically linked to established biomedical ontologies, limiting interoperability for those fields.

Third, although the benchmark spans 46 countries, the sampling strategy, which was weighted by author affiliation frequency in the LungCancer-OA subset, reflects existing publication biases and does not correct for the underrepresentation of some countries in the indexed literature. This affiliation-based weighting was used because patient-origin country was unavailable as a sampling criterion prior to manual curation; future benchmark expansions should incorporate deliberate oversampling of underrepresented regions, ideally guided by observed patient-origin distributions, to better support globally equitable extraction models.

Fourth, curation was restricted to the PMC Open Access subset, excluding cancer genomics articles available only behind paywalls. This likely introduces a systematic bias toward open-access publishing practices, which are not uniformly distributed across countries or research institutions.

Finally, the corpus was constructed through dual independent annotation followed by third-reviewer adjudication, and the reported inter-annotator agreement was calculated on the independent first-pass reviews prior to this adjudication. These agreement statistics therefore represent a conservative lower bound on the reliability of the gold standard dataset, as the disagreements they capture were subsequently resolved. We note two limitations of the annotation process: agreement was computed following dataset construction, rather than pre-specified as a study output, and structured calibration sessions were not conducted prior to annotation. The latter may have contributed to the moderate agreement observed on complex judgment fields such as genomic sequencing presence and study-aim classification. Future iterations of the benchmark should incorporate pre-annotation calibration to further strengthen consistency on these complex fields.

### 4.4 Literature mining and consortia: complementary approaches

A critical insight from our work is that literature mining and consortium-based approaches are complementary, not competitive. Consortia like TCGA and cBioPortal provide gold-standard molecular data with rigorous quality control, standardized formats, and rich clinical annotations, advantages that literature-mined data cannot match in terms of granularity and reliability. However, consortia’s geographic limitations and selective inclusion criteria create systematic coverage gaps. Hybrid integration models offer a path forward. Literature mining can identify underrepresented populations and candidate studies for consortium inclusion, serving as a discovery layer atop curated resources. For example, our identification of 96 studies with molecular data for 263 472 patients, none contributed to major consortia, reveals substantial untapped data sources. Automated extraction could flag these studies for expert review and structured data curation, systematically expanding consortium coverage. Collaborative annotation platforms engaging researchers from low- and middle-income countries can refine AI training datasets to better capture local molecular and environmental contexts. Linking geolocation metadata to environmental exposure databases (e.g. air quality indices, dietary surveys) can unravel gene-environment interactions critical to understanding regional cancer disparities.

Integration of AI-driven text mining with geolocation metadata reveals critical and previously overlooked molecular diversity within underrepresented populations, directly challenging the prevailing consortium-centric paradigm of precision oncology. For instance, the identification of a novel NRAS D54N mutation in primary lung melanoma from a Japanese cohort underscores the ability to detect rare, region-specific oncogenic drivers absent from TCGA and ICGC (Hibiya *et al.* 2020). Similarly, epigenetic regulation of the EWS-FLI1 fusion in Colombian Ewing’s sarcoma highlights molecular mechanisms pivotal to tumor biology in populations excluded from global databases (Montoya *et al.* 2020). In China, ERBB2 exon 16 skipping emerged as a resistance mechanism to EGFR inhibitors, illustrating splicing variants prevalent in Asian cohorts but overlooked in Western-centric datasets (Shi *et al.* 2020). Functional diagnostics in Finnish patients with rare epithelial-myoepithelial carcinoma demonstrated lineage-specific drug sensitivities, advocating for personalized strategies in cancers with limited representation (Mäkelä *et al.* 2020). Addressing these challenges through integrated, cross-disciplinary methods will move the field beyond merely cataloging geographic diversity to meaningfully leveraging it as a foundation for equitable, globally representative precision oncology. While resources such as the Human Ethnic and Regional Omics Database (HEROD) have demonstrated the value of ethnically and regionally annotated cancer genomics data, they remain limited to manually curated gene expression datasets rather than the comprehensive, automated extraction of diverse molecular data that our literature-mining approach enables (Zeng *et al.* 2017). AI-driven text mining is not a replacement for consortia but a vital partner, filling gaps in population coverage and environmental context that current resources alone cannot address. Together, these approaches ensure that molecular insights and therapeutic strategies reflect the full spectrum of global cancer biology, bridging the divide between localized research and consortium-driven precision medicine.

These findings extend beyond cancer genomics. The domain adaptation challenges, affiliation-origin discrepancies, and benchmark evaluation scripts we present apply broadly to biomedical literature mining across diseases, environmental health, and epidemiology. Future work should expand the benchmark to additional cancer types and non-cancer domains, implement the proposed multi-task learning architectures, and integrate extraction outputs with consortium curation workflows. Such systematic approaches will transform biomedical literature from underutilized archives into actively curated, globally representative data resources that advance both computational methodology and clinical equity.

## 5 Conclusion

Literature mining offers a scalable strategy for addressing critical geographic biases in cancer genomics by capturing molecular and geographic diversity excluded from major cancer consortia datasets. Our benchmark demonstrates that advanced AI geolocation tools are needed to systematically link molecular data with precise geographic and ancestry information, enabling separation of environmental exposures [e.g. aflatoxin-driven TP53 mutations in Sudan (Gouas *et al*. 2009), Guatemala (Alvarez *et al.* 2020), and Nigeria (Igetei *et al.* 2008)] from hereditary genomic variations (e.g. EGFR alterations in East Asian lung cancers) (Shi *et al.* 2020), in a reproducible way. This resource provides the training data and evaluation framework required to refine such models and quantify their performance across heterogenous articles and writing styles.

By advancing well validated AI-driven tools that capture environmental and ancestry-linked signatures, the field can ensure that downstream precision oncology applications better reflect the full spectrum of global cancer biology. We have established a robust benchmark dataset, with open-source code and FAIR-compliant disposition, that enables rigorous training and evaluation of future AI-driven approaches for geolocation and molecular data extraction in cancer research. This benchmark framework is designed to address systemic biases in existing datasets and to support integration of literature-mined outputs with consortium resources and other biomedical data platforms, laying methodological groundwork for more globally representative analyses.

## Supplementary Material

vbag225_Supplementary_Data

## Data Availability

The data underlying this article are available in *Zenodo* at *zenodo.org*, and can be accessed with DOI 10.5281/zenodo. The dataset is licensed under Creative Commons Attribution 4.0 International (CC-BY 4.0), permitting unrestricted use with attribution, and is designed to support benchmark extension by the community.
