# Supplementary material for: A FAIR gold-standard benchmark for geographic entity recognition in cancer genomics literature for biomedical NLP
Source: Bioinform Adv. 2026 Aug 27;6(1):vbag225. doi: 10.1093/bioadv/vbag225 (PMC13575932; doi:10.1093/bioadv/vbag225)
Supplement: Supplementary file 1 [file vbag225_supplementary_data.docx]

**Supplementary File 1: PubMed queries for cancer genomics and lung cancer literature mining.** Full search strategies used to retrieve manuscripts on “cancer genomics” and “lung cancer genomics,” including all Boolean operators and MeSH terms, for the manual curation of the benchmark dataset.

1-PubMed query used to retrieve “cancer genomics” manuscripts:

("cancer*"[All Fields]

OR "neoplasms"[MeSH Terms] OR "neoplasm*"[All Fields]

OR "tumor*"[All Fields] OR "tumour*"[All Fields]

OR "neurofibroma*"[All Fields]

OR "carcinoma*"[MeSH Terms] OR "carcinoma*"[All Fields]

OR "sarcoma*"[All Fields] OR "leukaemia*"[All Fields] OR "leukemia*"[All Fields] OR "lymphoma*"[All Fields] OR "myeloma*"[All Fields] OR "oncolog*"[All Fields] OR "malignan*"[All Fields] OR “pan-cancer” [All Fields] )

AND (

"genetic phenomena"[MeSH Terms]

OR "genome"[MeSH Terms] OR "genome*"[All Fields]

OR "genomics"[MeSH Terms] OR  "genomic*"[All Fields]

OR "mutation"[MeSH Terms] OR “mutate*"[All Fields]  OR “mutati*"[All Fields]  OR “mutato*"[All Fields]

OR "genetics"[MeSH Subheading] OR "genetic*”[All Fields]

OR "genetic techniques"[MeSH Terms] OR "genetic techniques"[All Fields]

OR "precision medicine"[MeSH Terms] OR "precision medicine"[All Fields]

OR "genetic therapy"[MeSH Terms] OR "genetic therapy"[All Fields]

OR  "transcriptome"[MeSH Terms] OR  "transcriptome"[All Fields]

OR "whole genome sequencing"[MeSH Terms]

OR "exome"[MeSH Terms] OR "exome"[All Fields]

OR "Proteomics"[MeSH Terms] OR "proteome"[MeSH Terms]  OR “proteome*”[All Fields]

OR "Sequence Analysis"[MeSH Terms] OR "Sequence Analysis"[All Fields]

OR "Genome-Wide Association Study"[MeSH Terms]

OR "High-Throughput Nucleotide Sequencing"[MeSH Terms] OR “sequencing”[All Fields]

OR "Molecular Sequence Data"[MeSH Terms] OR “Sequence data”[All Fields]

OR "Oligonucleotide Array Sequence Analysis"[MeSH Terms]

OR "DNA Mutational Analysis"[MeSH Terms]

OR "Gene Expression Profiling"[MeSH Terms] OR “Gene Expression”[All Fields]

)

AND 2000/01/01:3000/12/31[Date - Publication]

2-PubMed query used to retrieve “lung cancer genomics” manuscripts:

("lung neoplasms"[MeSH Terms]

OR ("lung"[All Fields] AND "neoplasm*"[All Fields])

OR ("lung"[All Fields] AND "cancer*"[All Fields])

OR ("lung"[All Fields] AND "carcinoma*"[All Fields])

OR ("lung"[All Fields] AND "tumour*"[All Fields])

OR ("lung"[All Fields] AND "tumor*"[All Fields])

OR ("pulmonary"[All Fields] AND "neoplasm*"[All Fields])

OR ("pulmonary"[All Fields] AND "cancer*"[All Fields])

OR ("pulmonary"[All Fields] AND "carcinoma*"[All Fields])

OR ("pulmonary"[All Fields] AND "tumour*"[All Fields])

OR ("pulmonary"[All Fields] AND "tumor*"[All Fields])

OR ("adenocarcinoma"[All Fields] AND "lung"[All Fields])

OR ("adenocarcinoma"[All Fields] AND "pulmonary"[All Fields])

OR "nsclc"[All Fields] OR "nsclc s"[All Fields] OR "nsclcs"[All Fields]

OR “pan-cancer”[All Fields])

AND (

"genetic phenomena"[MeSH Terms]

OR "genome"[MeSH Terms] OR "genome*"[All Fields]

OR "genomics"[MeSH Terms] OR  "genomic*"[All Fields]

OR "mutation"[MeSH Terms] OR “mutate*"[All Fields]  OR “mutati*"[All Fields]  OR “mutato*"[All Fields]

OR "genetics"[MeSH Subheading] OR "genetic*”[All Fields]

OR "genetic techniques"[MeSH Terms] OR "genetic techniques"[All Fields]

OR "precision medicine"[MeSH Terms] OR "precision medicine"[All Fields]

OR "genetic therapy"[MeSH Terms] OR "genetic therapy"[All Fields]

OR  "transcriptome"[MeSH Terms] OR  "transcriptome"[All Fields]

OR "whole genome sequencing"[MeSH Terms]

OR "exome"[MeSH Terms] OR "exome"[All Fields]

OR "Proteomics"[MeSH Terms] OR "proteome"[MeSH Terms]  OR “proteome*”[All Fields]

OR "Sequence Analysis"[MeSH Terms] OR "Sequence Analysis"[All Fields]

OR "Genome-Wide Association Study"[MeSH Terms]

OR "High-Throughput Nucleotide Sequencing"[MeSH Terms] OR “sequencing”[All Fields]

OR "Molecular Sequence Data"[MeSH Terms] OR “Sequence data”[All Fields]

OR "Oligonucleotide Array Sequence Analysis"[MeSH Terms]

OR "DNA Mutational Analysis"[MeSH Terms]

OR "Gene Expression Profiling"[MeSH Terms] OR “Gene Expression”[All Fields]

)

AND 2000/01/01:3000/12/31[Date - Publication]

**Supplementary Figure 1: Flowchart of Study Screening and Selection.**

Flowchart illustrating the manual curation process for the benchmark dataset. Of 300 potentially relevant manuscripts, exclusions were made for non-cancer focus, absence of human subjects, and lack of reported geographic location, resulting in 129 cancer research manuscripts with geographic data (91 on lung cancer, 22 on other cancers, and 16 on both).

**Supplementary Table 2: GeoBoost2 Performance Metrics Against Gold Standard (n=129 articles)**

| **Metric Type** | **Precision** | **Recall** | **F1-Score** | **TP** | **FP** | **FN** |
| --- | --- | --- | --- | --- | --- | --- |
| Macro (per-article avg) | 35.30% | 76.20% | 55.30% | - | - | - |
| Micro (per-country, n=449) | 32.90% | 74.70% | 45.70% | 133 | 271 | 45 |
| **Article-level summary** |  |  |  |  |  |  |
| >=1 correct country | 79.1% (102/129) |  |  |  |  |  |
| Perfect extraction | 7.0% (9/129) |  |  |  |  |  |
| Any false positives | 89.1% (115/129) |  |  |  |  |  |

*Macro metrics average precision/recall/F1 across 129 articles independently.

*Micro metrics aggregate all country predictions (TP=133, FP=271, FN=45).

Per-article CSV available in dataset repository.

**Supplementary Table 1:** **Manuscripts with patient origins not captured by author affiliations.** List of PubMed IDs (PMIDs) for manuscripts in which the gold standard curation identified patient cohort countries not represented in the author affiliations. This highlights instances where patient geographic origins were missed by affiliation-based metadata extraction.

| **PMID** | **Gold Standard not in Affiliation** |
| --- | --- |
| 32858747 | United States |
| 37298323 | United States |
| 38503267 | Italy, Norway |
| 35323374 | Japan, SE Asia |
| 35194111 | Germany |
| 37641131 | Germany |
| 30473379 | France |
| 32159882 | China, Hong Kong, Italy, Poland, South Korea |
| 35830375 | Canada, Ireland |
| 36347854 | Canada |
| 33434335 | Australia, Canada, Italy, Philippines |

**Supplementary Table 2.** **Geographic representation of eight major cancer genomic databases.** Summary of key cancer genomics resources evaluated in this study, including main host country and affiliated participating countries (where applicable). See manuscript Table/Figure references as appropriate.

| **Database Name** | **Main Host (Secretariat)** | **Other Countries Involved (Collaborations)** |
| --- | --- | --- |
| TCGA | USA | USA |
| cBioPortal | USA | USA, China, France, Japan, UK, Singapore, Italy, S. Korea |
| COSMIC | UK | UK, USA (ISB) |
| ICGC | Canada | Australia, Canada, China, France, Germany, India, Japan, |
|  |  | Singapore, UK, USA, S. Korea, Spain, Italy, Mexico, Saudi Arabia |
| GDC | USA | USA |
| UCSC Xena | USA | USA |
| OncoDB | USA | USA |
| AACR GENIE | USA | USA, Canada, France, Netherlands |

**Supplementary File 2.**

**Data extraction form used for manual curation.** Complete standardized extraction form provided to curators for systematic data collection, covering study location, disease information, cohort size, patient demographics, molecular data, clinical trial participation, and linked repository information.

**General information**

Title of paper / abstract / report that data are extracted from

**Study ID:**

**Title:**

**Inclusion Check**

The next two questions are to determine if the study is one of interest. If either question is negative (i.e., not cancer OR not human subjects) , no further curation of the paper is needed.

**Disease**

**Disease Studied**

Select the disease the study is focused on. If other disease (not cancer related), no other information should be extracted from paper.

- Lung Cancer
- Other Cancer
- Other Disease (not cancer related)
- Other:

If 'Other Cancer' is selected above, please enter the name of the cancer(s) studied.

Separate multiple cancer mentions with a semicolon (;)

**Humans Subject Research**

Does the study include human subjects or data from human subjects?

If no is selected, no other information should be extracted from the paper.

- Yes
- No

**Geographic Location of Participants**

Is the geographic location of the participants, or cohort, included of the study reported in the paper?

If 'No' is selected, skip the next question

- Yes
- No

**Notes**

Add any notes with relevant information (e.g., analyzed data from a repository), if needed.

Where was the geographic location information found in the paper

Select all the sections of the paper where the geographic (location) information of the patients was found.

- Title
- Text
- Table(s)
- Figure(s)
- Supplementary Material
- Other:

**Country in which the participants are located:**

Select the location(s) of the participants included in the study. The top 15 countries, based on study affiliations, are listed in alphabetical order for easy selection. Please select 'Other' for any participant location not on this list. Enter the other country, or countries, separated by a semicolon (;).

- Australia
- Canada
- China
- France
- India
- Israel
- Italy
- Japan
- Netherlands
- Singapore
- Spain
- Sweden
- Switzerland
- UK
- USA
- Other:

**Evidence Sentence(s)**

In this field, please cut and paste the sentence(s) containing evidence for the location(s) of the participants selected above. If the evidence is from a table or figure, cut and paste the table or figure title.

**Table preview**

For studies with multiple cohorts in multiple locations (by country), complete the table with the name of the location and the number of participants in that location. If there are more than 5 locations, enter the remaining countries in row 6, column 1(comma separated) with their respective counts in column 2

|  | Location (Country) Name | Number of Participants |
| --- | --- | --- |
| Location #1 |  |  |
| Location #2 |  |  |
| Location #3 |  |  |
| Location #4 |  |  |
| Location #6 |  |  |
| Location #6+ |  |  |

**Characteristics of included studies**

**Is the study a clinical trial?**

- Yes
- No
- Other

If yes, please enter the Clinical trial registration number:

Methods

**Aim/focus of study**

- Diagnosis
- Treatment
- Outcome
- Genomic (e.g., GWAS, variants)
- Disease Prediction
- Other

**Total number of participants**

Enter the total number of participants included in the study.

**Number of cohorts**

Enter the number of cohorts. These could be cohorts form different locations sites or case/control cohorts.

**Demographic Information**

**Is any demographic information about the participants reported?**

This information can be reported either at an individual level or as summary statistics of the cohort. This information may not be accessible from the PDF, the full article online should be checked for data availability or supplemental files.

- Age
- Sex/Gender
- Race/Ethnicity/Nationality
- No demographic information

**Location in the manuscript of the demographic information:**

- Text
- Table(s)
- Figure(s)
- Supplemental Material

**Is the demographic information reported in aggregate or by individual**

- Individual
- Aggregate
- Other

**Baseline Population Characteristics**

For race/ethnicity/nationality, enter the specifics of the reported information with the R/E/N in column 1 and the number of participants in that group in column 2.

|  | R/E/N | Number of Participants |
| --- | --- | --- |
| R/E/N 1 |  |  |
| R/E/N 2 |  |  |
| R/E/N 3 |  |  |
| R/E/N 4 |  |  |
| R/E/N 5 |  |  |
| R/E/N 6 |  |  |
| R/E/N 7 |  |  |
| R/E/N 8 |  |  |
| R/E/N 9 |  |  |
| R/E/N 10 |  |  |

**Mutation Data**

**Does the study report any genomic mutation information about the participants?**

**If the answer is No, skip the next two questions**

- Yes
- No
- Other

**If yes, where in the paper is this information reported:**

- Text
- Table(s)
- Figure(s)
- Supplemental Material
- Other:

**Is the information reported by individual or in aggregate**

- Individual
- Aggregate
- Other

**Genomic Data**

**Does the study include any genomic sequencing data from the participants?**

This information may not be accessible from the PDF, the full article online should be checked for data availability or supplemental files.

- Yes
- No
- Other

**If yes, where in the paper is this information reported?**

- Text
- Table(s)
- Figure(s)
- Supplemental Material
- Data Availability Statement
- Other

**Is the genomic data reported by individual participant or in aggregate?**

- Individual
- Aggregate
- Other

**Does the study report that the genomic information was deposited into a database or repository?**

- Yes
- No

**If yes, please enter the repository name followed by any ids/accession numbers given (for example** **- dbGaP: phs002192)**

**Notes**

**Additional Information**

Use this field to enter any additional information such as any questions about or difficulty encountered with the extraction, or any information not captured in the extraction form that you feel may be relevant .

**Supplementary File 3**

**Curator training guidelines.** Detailed description of the guidelines and protocols used to train curators for standardized data extraction. These instructions ensured that, for each article, two curators independently extracted all relevant data, with a third curator resolving disagreements and finalizing the gold standard dataset.

For this project, we will be extracting the location information of participants in cancer related studies, as was as some other pertinent information in the study. This document provides explanations and instructions for the extraction.

## Curation tool

For the curation task, we will use the Covidence tool. This is a web-based app that can be accessed at: <https://app.covidence.org/reviews/467005>. All curators will receive an email invitation that will grant them access to the project.

Once the project is open, curators should select ‘Continue’ in the Extraction box. This will present to the curator articles available for extraction. The curator should select “Begin Extraction” to start the extraction of information from the article.(see Figure 1)

Figure 1: Screenshots of Covidence showing the steps for accessing an article to begin extraction.

After a study is selected, a pdf of the paper will be shown on the left with the data extraction panel on the right (Figure 2). The remainder of this document will provide the instruction for completing the data extraction for each paper.

Save or Finish

Extracted data entry

PDF

Header

Figure 2: Extraction interface in Covidence

## Extraction Instructions

The following provides instructions for completing the data extraction sections for each paper. Where further explanation may be warranted, the instructions will give the data field and the information that should be provided in each field.

### Study information Section

This section extracts information about the paper from which the data is being extracted. While this information may seem redundant it is required as after extraction is competed the data from Covidence is exported to a data frame and these fields are necessary to identify the study associated with the data.

**Study ID**: the first author’s last name and year of study, this can be copied from the study header (Figure 2).

**Title**: the title of the article

### Inclusion Check

In this section, the curator will check that this study is in fact on that should be included for further data extraction. For this project, we want to extract relevant information from studies related to lung cancer AND include human participants in the study. While we have selected these articles based on a search related to lung cancer, there is a chance they were selected due to a mention of lung cancer though it may not be the focus of the study. Because we are interested in data related to participants of the study, we only want to include study that have human participants.

**Disease Studied**: curator should select the disease the study is focused on. If other cancers, alone or in addition to lung cancer are studied, the names of those cancers should be entered in the text field following the question.

**Human Subjects Research**: curator should select yes, if the study included human participants, or used data, such as downloaded genomic data, from human participant. While some articles may discuss the cancer in humans, if no participants were included in the study, ‘no’ should be selected.

Note, both criteria must be met to include the study for further information extraction. If either the disease studied is not cancer OR there are no human subjects included in the study, the data extraction is complete for this article and the curator can complete the document.

### Geographic Location of Participants

In this section, the curator will assess whether participant geographic location information is provided in the article. If so, the curator will extract the reported geographic location of all participants. Note, the location information should be extracted at the country level.

**Supporting sentences**: curators should extract all supporting sentences that report the location of the participant

**Identifying section within the paper where the geographic information is reported**. Curators will also identify where in the paper the geographic information is reported (title, text, tables, figures, and/or supplemental material.

**Table for multiple participant locations:** For studies that contain multiple geographic locations, the curators should complete the table identifying the number of participants in each geographic location.

**Notes**: This section also includes a Notes field, which is not required but can be used if the curator feels there is some addition information regarding the location of the participants that should be recorded.

### Characteristics of included study

This section includes some additional information about the study or its participants that should be extracted. Note, some of this information may not be available in the pdf, the curator should check the article either in PubMed or the journal site for the reporting of this information.

**Clinical Trial**: classification of study as Clinical trial or not. If yes, the registration number of the clinical trial will be entered

**Aim/focus of study**: the main aim or focus of the study should be selected. These may include the diagnosis, treatment or outcome of the cancer, or a focus on the genomics of the participants. Multiple selections can be made as needed.

**Number of Participants**: the total size of the cohort studies should be entered

**Number of Cohorts**: the number of different patient cohorts included should be entered

**Demographic information**: for this, just the presence or absence of the demographic information should be extracted by checking the box next to the relevant information. Multiple boxes can be checked for each of the specific demographic information listed or their location in the article. ‘No’ should be selected only if none of the 3 types of demographic information is reported.

**Identifying section within the paper where the demographic information is reported**. Curators will also identify where in the paper the demographic information is reported (title, text, tables, figures, and/or supplemental material).

**Classification of reporting level**: Curators will classify whether the demographic information is reported at the individual or aggregate level.

**Table for race/ethnicity/nationality:** For studies that report the R/E/N of participants, the curators should complete the table identifying the number of participants in each R/E/N class.

### Genomic sequencing and mutation data:

**Mutation data**: the curator will first identify if any mutation data of the participants is reported. If there is none, no further extraction is required. If there is mutation data, the curator will:

**Identifying section within the paper where the mutation data is reported**. Curators will also identify where in the paper the demographic information is reported (title, text, tables, figures, and/or supplemental material).

**Classification of reporting level**: Curators will classify whether the mutation information is reported at the individual or aggregate level.

**Genomic data**: the curator will first identify if any genomic sequencing data of the participants is reported. If there is none, no further extraction is required.

**Identifying section within the paper where the genomic data is reported**. Curators will also identify where in the paper the demographic information is reported (title, text, tables, figures, and/or supplemental material).

**Classification of reporting level**: Curators will classify whether the gemomic information is reported at the individual or aggregate level.

**Database/Repository Information**: the curator should note is this data has been deposited to a repository and if so, should extract the repository name and any identifier associated with the data.

### Notes

This final section is not required but is available for curator to make notes related to the curation of the article, including any difficulties or questions encountered during curation.

## Completion of Extraction

After all data has been extracted, the curator should select “send for consensus” at the top right (Figure 2). If the curator needs to exit from the article or the app before the extraction is completed, they should select “Save” before exiting.
